# Ligand–Enzyme Interaction Modeling of Missense Variants Implicated in Mitochondrial HMG-CoA Synthase Deficiency

**DOI:** 10.3390/ijms26178266

**Published:** 2025-08-26

**Authors:** María Arnedo, David Ros-Pardo, Beatriz Puisac, Cristina Lucia-Campos, Marta Gil-Salvador, Ana Latorre-Pellicer, Íñigo Marcos-Alcalde, Juan Pié, Paulino Gómez-Puertas

**Affiliations:** 1 Unit of Clinical Genetics and Functional Genomics, Department of Pharmacology and Physiology, School of Medicine, University of Zaragoza, CIBERER and IIS-Aragon, 50009 Zaragoza, Spain; marnedo@unizar.es (M.A.); puisac@unizar.es (B.P.); alatorre@unizar.es (A.L.-P.); juanpie@unizar.es (J.P.); 2 Molecular Modeling Group, Centro de Biología Molecular Severo Ochoa, CSIC-UAM, C/ Nicolás Cabrera 1, 28049 Madrid, Spain; dros@posta.unizar.es (D.R.-P.); imarcos@cbm.csic.es (Í.M.-A.)

**Keywords:** HMG-CoA synthase, mHS STAG2, mHS deficiency, molecular modeling, variant rationalization

## Abstract

Human mitochondrial 3-hydroxy-3-methylglutaryl-CoA synthase (HMG-CoA synthase, mHS) synthase is a key enzyme in ketogenesis and is located mainly in the liver, but also in the colon, skeletal muscle, heart, pancreas, and testes. It is an inner mitochondrial membrane-associated protein. Mutations in the *HMGCS2* gene, which encodes this enzyme, lead to “mHS deficiency,” a rare, autosomal recessive, inherited metabolic disorder. To date, about 100 patients with this disorder have been described. The disorder usually appears during the first year of life, often after a period of starvation or an intercurrent illness. A total of 77 different DNA mutations has been described that are considered responsible for mHS deficiency, although the mechanisms leading to loss of function are not fully understood. To study how the different missense variants affect the enzymatic activity of the protein on an atomic scale, we used molecular dynamics computational simulation techniques for variants whose activity could be measured “in vitro.” The study included a total of 46 molecular dynamics trajectories of enzyme–substrate/product interaction simulations, each 500 ns long (23 microseconds total). Currently, the atomic and biophysical effects of the mHS variants on their catalyzed reactions have not been studied in detail experimentally. To our knowledge, molecular dynamics simulations are one of the most promising tools for understanding the molecular basis of the phenotypic consequences of these variants. In the present work, molecular dynamics simulations reliably reproduce most experimental enzyme activity measurements, supporting their future application to the study of new mHS mutations.

## 1. Introduction

**Figure 1:**
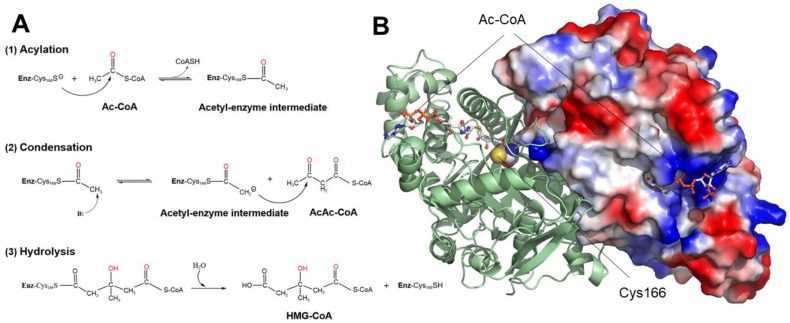
Enzymatic reaction catalyzed by the enzyme human mitochondrial HMG-CoA synthase (mHS). (**A**) Schematic of the three phases (acylation, condensation, and hydrolysis) of the reaction taking place in the active center of the enzyme, from the initial substrate acetyl-CoA (Ac-CoA) and the intermediate substrate aceto-acetyl-CoA (AcAc-CoA) to the final product 3-hydroxy-3-methylglutaryl-CoA (HMG-CoA) through the intermediate phases of the acetylated enzyme at the SH group of Cys166 (Enz-Cys166S). (**B**) Position of two initial substrate molecules, acetyl-CoA (Ac-CoA), in the substrate tunnels of the mHS homodimer. One monomer is depicted as a surface, and the other is depicted as a ribbon showing the secondary structure. The location of the catalytic residue Cys166 in the active center of one monomer is indicated.

Mitochondrial 3-hydroxy-3-methylglutaryl-CoA synthase (HMG-CoA synthase, mHS, *HMGCS2*, EC 2.3.3.10) is a key enzyme in ketogenesis, the metabolic pathway that generates ketone bodies (acetoacetate, β-hydroxybutyrate, and acetone) from fatty acids [1]. The most recognized physiological role of ketone bodies is to serve as an alternative energy source to glucose [2]. Currently, there is growing interest in their functions as cell signaling molecules, as well as in their epigenetic and post-translational modulation properties [3]. mHS is a 508-amino acid protein associated with the inner mitochondrial membrane, primarily located in the human liver and colon but can also be found in the skeletal muscle, heart, pancreas, and testes [4,5]. This enzyme catalyzes the condensation reaction of acetyl-CoA (Ac-CoA) and acetoacetyl-CoA to form 3-hydroxy-3-methylglutaryl-CoA (HMG-CoA). This reaction follows a three-step Bi Bi Ping-Pong substitution mechanism (Figure 1A). The first, rate-limiting step is an acylation reaction, where a cysteine residue in the enzyme’s active site acts as a nucleophile on the carbonyl group of acetyl-CoA to form an acetyl–enzyme intermediate. The second step is a condensation reaction involving a nucleophilic attack on the second substrate, acetoacetyl-CoA, which leads to HMG-CoA formation. In the third step, this product is released by hydrolysis [6]. Studies of the enzyme’s crystallization confirmed that it is active as a homodimer, with two oppositely oriented monomers occupying an interface, accounting for 15% of the total molecular surface area [7,8] (Figure 1B).

Mutations in the *HMGCS2* gene (NCBI GenBank NM_005518.4; Gene ID 3158) which encodes this enzyme, lead to mHS deficiency. This is a rare metabolic disorder with an autosomal-recessive inheritance (OMIM 600234) [9]. To date, around 100 patients have been reported [10]. It typically appears during the first year of life, often following a period of starvation or intercurrent illness. Initial symptoms include vomiting, altered consciousness, and hepatomegaly [9]. Most patients present with acute metabolic decompensation, characterized by hypoglycemia, dicarboxylic aciduria, and other metabolites in the urine, such as 4-hydroxy-6-methyl-2-pyrone (4HMP), 3-hydroxydicarboxylic, and 5-hydroxyhexanoic acids [11]. Diagnosis is challenging due to the non-specific clinical and biochemical presentation.

In view of the difficulties of confirming the diagnosis through a liver biopsy, a method for the expression and measurement of enzyme activity was developed and applied to the analysis of missense mutations [12,13]. Although, in most cases, the activity was less than 10%, it reached values of 30% and 70%, respectively, for the F174L and R505Q variants [12,13,14]. In this regard, it was shown that missense mutations exhibited an irregular distribution and that most were located far from the substrate cavity, suggesting that the dimerization surface was crucial in the active site structure [13].

These findings raise questions about the molecular mechanisms by which these mutations cause the disease. In the present work, we will address the computational simulation of different mutations for which their enzymatic activity has been experimentally determined and will try to provide an explanation of the effect of the mutations on the stability in the substrate channel of both the initial substrate and the final product of the reaction.

## 2. Results and Discussion

The mHS protein is structurally arranged as a homodimer (PDB id: 2WYA; [7]) in which the substrate tunnels corresponding to both monomers are located at opposite ends and in which the maintenance of the structure of the active center depends not only on the proper structure of each monomer but also on the correct arrangement of the opposite monomer.

To date, a total of 77 different mutations has been described in the *HMGCS2* DNA and is considered responsible for mHS deficiency. These mutations are summarized in Table 1, which was compiled from all the papers published to date. Two correspond to defects in splicing mechanisms leading to exon 1 deletion, while six others result in aberrant splicing in the mHS protein. Moreover, 21 of the aforementioned mutations are point mutations that lead to the appearance of premature stop codons, either directly or as a result of a shift in the reading phase of the gene. In all these cases, the result is an incomplete protein unable to perform normal enzymatic activity. The remaining 48 mutations described give rise to 47 different amino acid changes (missense mutations) whose effect on enzyme activity may be related to changes in the structure of the active center or substrate placement site, to changes at the dimerization interface, or to other putative changes in the protein structure.

**Figure 2:**
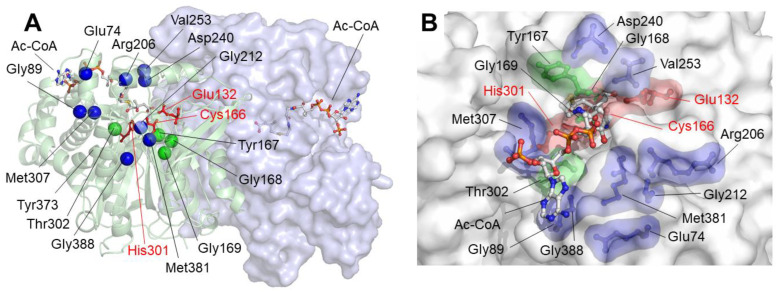
Position of missense variants predicted to affect active center and substrate tunnel. (**A**) The position of the alpha carbon of amino acids that correspond to variants that are potentially involved in the activity of the active center is shown in green. The position of amino acids that are located around the substrate binding site is shown in blue. The residues of the catalytic triad (Glu132, Cys166, and His301) are depicted as red sticks. The location of the acetyl-CoA (Ac-CoA) molecules is also indicated. (**B**) Details of the orientation of an Ac-CoA molecule in the substrate entry tunnel. The positions of the same amino acids as in panel A are indicated with the same color coding.

**Figure 3:**
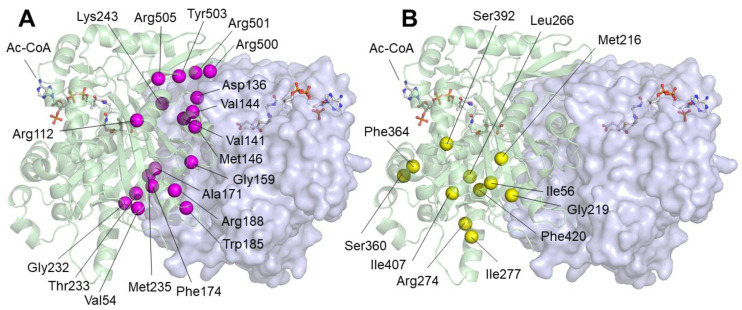
Position of missense variants in mHS dimer predicted to affect dimerization or located far from the active center or the homodimerization surface. (**A**) The position of the alpha carbon of amino acids corresponding to variants that are potentially involved in maintaining dimer stability is indicated by magenta spheres. (**B**) The alpha carbons of amino acids corresponding to variants located distally from the active center or dimerization surface are shown as yellow spheres.

Figure 2 and Figure 3 show the locations of the amino acids involved in missense variants. The amino acids are grouped according to their positions in the protein structure, with the exception of the residue Arg35 (R35M variant) that localizes in an area not covered by the crystallized structure of the protein [7]. The first cluster is composed of residues located in the vicinity of the active center of the protein: Tyr167, Gly168, Gly169, and Thr302, colored green in Figure 2. These amino acids are located close to the residues of the catalytic triad: Glu132, Cys166, and His301, colored red in Figure 2. The second group of amino acids is composed of those that, without intervening directly in the enzymatic reaction, are located in the vicinity or participate in the correct localization of the substrate in the cavity of the active center: Glu74, Gly89, Arg206, Gly212, Asp240, Val253, Met307, Met381, and Gly388, colored blue in Figure 2.

A third group of amino acids correspond to those that, because their location close to the monomer–monomer interface, can influence the homodimerization of the enzyme. The correct dimer structure is critical for the maintenance of the geometry of the active center [7]. Residues corresponding to this group are represented as magenta spheres in Figure 3A: Val54, Arg112, Asp136, Val141, Val144, Met146, Gly159, Ala171, Phe174, Trp185, Arg188, Gly232, Thr233, Met235, Lys243, Arg500, Arg501, Tyr503, and Arg505 belong to this group. The last group of residues, represented as yellow spheres in Figure 3B, is composed of those located far from the active center or the homodimerization surface: Ile56, Met216, Gly219, Leu266, Arg274, Ile277, Ser360, Phe364, Ser392, Ile407, and Phe420. The role of mutations in these residues in protein activity may be related to changes in global geometry that are somehow transmitted to the active center.

To study the effect that different missense variants could have on the enzymatic activity of the protein at the atomic scale, we used computational molecular dynamics simulation techniques. We studied the interaction dynamics of the enzyme substrate (Ac-CoA) and the final reaction product (HMG-CoA) with the tunnel forming the active center. The present study has not included the interaction dynamics of the intermediate substrates, including the acetyl–enzyme complexes with the substrate acetocetyl-CoA due to the complexity of this simulation. Also, in all cases, due to the symmetry of the system, the interaction of the substrate or product with only one of the two active centers has been taken into account. Only variants for which an experimental assay of enzyme activity has been performed were considered in the simulations (see Table 1 and Table 2). The remaining variants were not simulated because it could not be ruled out that the effect of the mutation was related to problems during protein folding rather than the nature of its interaction with the ligands. With these simplifications included, the study resulted in the simulation of 46 interactions in molecular dynamics trajectories, each 500 ns long (23 microseconds total): twenty-two variants and the wild-type protein were simulated in the presence of the substrate Ac-CoA or the final product HMG-CoA.

For the wild-type protein, we placed either an Ac-CoA or a HMG-CoA molecule in the active center, starting from the position closest to that of the substrates in the crystallized enzyme [7]. After that, following the procedures described in the Methods Section, we carried out a series of steps: solvation in explicit simulated water, minimization of the structure, gradual equilibration up to a temperature of 37 °C, and final stabilization by eliminating all positional restrictions. Then, the systems were subjected to 500 ns trajectories of molecular dynamics simulation, during which the positions of the ligands and amino acids in the protein were measured. The structural movements of the system were monitored by measuring the root mean square deviation (RMSD). During the 100–500 nanosecond interval, the variation in RMSD values was consistently less than 1.5 Angstrom, which is indicative of structural stability (Supplementary Figure S1).

**Figure 4:**
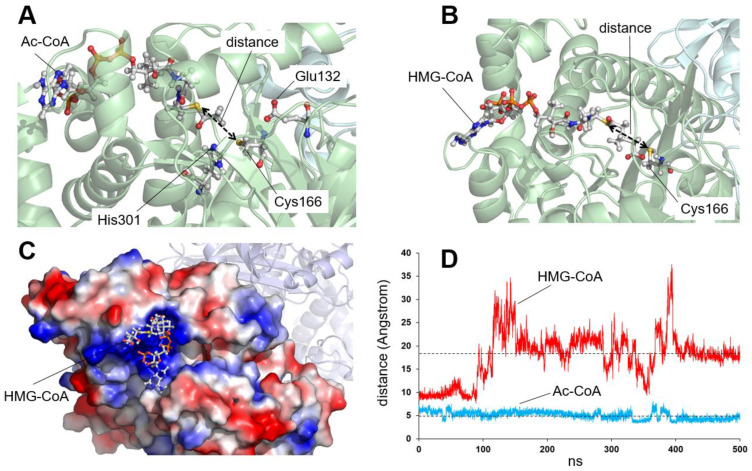
Analysis of the stability of an acetyl-CoA (Ac-CoA) molecule or a HMG-CoA molecule in the active center of wild-type mHS. (**A**) Schematic of the position of an Ac-CoA molecule in the active center of the wild-type enzyme prior to molecular dynamics simulation. The residues of the catalytic triad (Glu132, Cys166, and His301) are depicted as sticks. The distance to be monitored between the sulfur atom of the amino acid Cys166 and the sulfur atom of the Ac-CoA molecule is indicated. (**B**) Schematic of the position of a HMG-CoA molecule in the active center of the wild-type enzyme prior to molecular dynamics simulation. The position of the catalytic amino acid Cys166 is shown. The distance to be monitored between the sulfur atom of Cys166 and the sulfur atom of HMG-CoA is indicated. (**C**) Localization of a HMG-CoA molecule on the surface of the mHS enzyme after 500 ns of molecular dynamics simulation. Note how the molecule has moved from its initial position (shown in panel B) to its final position outside the substrate tunnel. (**D**) Plot showing the variation in the distance (in Angstrom) between the sulfur atom of the amino acid Cys166 and the sulfur atom of Ac-CoA (blue line, stable) or HMG-CoA (red line, unstable) molecules, measured over 500 ns of molecular dynamics simulation trajectory. The dashed lines indicate the average values measured for the two distances over the final 300 ns of the simulation (4.9 and 18.5 Angstrom, respectively). These lines are included in the other figures for comparison purposes.

Figure 4 illustrates the behavior of an Ac-CoA substrate molecule and a HMG-CoA product over the course of a 500 ns simulation. To monitor the position of the ligands, we chose to measure the distance between the sulfur atom of the acetyl group of Ac-CoA or HMG-CoA and the sulfur atom of the catalytic Cys166 (Figure 4A,B). These two atoms are involved in the acylation and hydrolysis steps of the enzymatic reaction and must stay close together for the reaction to start. Therefore, this distance is indicative of the correct position of the substrate or the final product within the enzyme’s active site. As shown in Figure 4D (violet line), during the simulation, the Ac-CoA molecule remains in the initial position without significant variation, which is consistent with the situation in which this substrate reacts with the enzyme to initiate the first step of the reaction. In contrast, the behavior of the HMG-CoA molecule is the opposite (Figure 4D, red line): after a few ns, the ligand separates from the active center and exits the substrate tunnel, now open (Figure 4C), and leaving it free for an Ac-CoA substrate to arrive and start the reaction again.

The real rate of the purified enzyme under laboratory conditions is around 1.2 molecules of substrate transformed per second and per protein molecule (0.83 s per molecule of substrate), as can be deduced from the reported experimental values of specific activity [12,13]. The simulation times used in the present work are certainly far from these values (0.5 microseconds versus 0.83 s) but may be sufficient to analyze the stability of each ligand in the active center, both in the wild-type protein and in the different variants. Indeed, the results obtained are consistent with the expected roles of the substrate, Ac-CoA, before the reaction and the product, HMG-CoA, after the reaction.

After establishing the behavior of both ligands in the wild-type protein, the process was repeated, under the same simulation conditions, for the missense variants, for which an experimental assay of enzyme activity has been published (see Table 1). Homology modeling procedures were used to replace the original amino acid with the variant residue for each mutated enzyme. Thus, 500 ns simulations were performed for the four groups of variants mentioned above: residues located in the active center or its vicinity, residues close to the ligand tunnel, residues close to the homodimerization surface, and a fourth group with the remaining variants (see Table 2).

### 2.1. Residues Located Close to the Active Center

**Figure 5:**
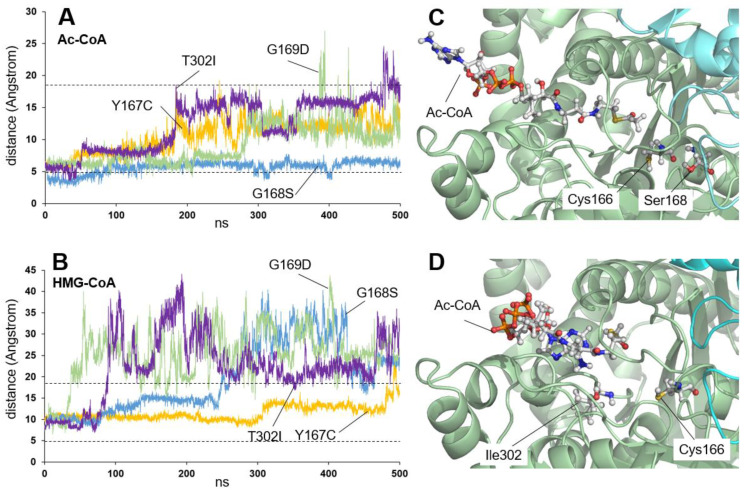
Analysis of the stability of acetyl-CoA (Ac-CoA) or HMG-CoA in mHS missense variants predicted to affect the active center. (**A**,**B**) Plot showing the variation in the distance (in Angstrom) between the sulfur atom of the amino acid Cys166 and the sulfur atom of Ac-CoA (**A**) or HMG-CoA (**B**) molecules, measured over 500 ns of molecular dynamics simulation trajectory of the four variants Y167C (yellow), G168S (blue), G169D (green), and T302I (violet). The dashed lines mark the average values measured for the wild-type protein (see Figure 1). (**C**) Representation of the stable position of an Ac-CoA molecule in the active center of the G168S variant after 500 ns of molecular dynamics simulation. The locations of the amino acids Cys166 (catalytic) and Ser168 (mutated) are indicated. (**D**) Schematic of the position of an Ac-CoA molecule away from the active center of the T302I variant after 500 ns of molecular dynamics simulation. The locations of the amino acids Cys166 (catalytic) and Ile302 (mutated) are indicated.

In the case of residues located in the active center (in green in Figure 2), Tyr167, Gly168, Gly169, and Thr302 are located next to the catalytic residues (Glu132, Cys166, and His301). In all four cases, a distortion of the catalytic center is predicted. Figure 5 illustrates the distance between the sulfur atom of the CoA moiety and the sulfur atom of Cys166 for the four variants (Y167C, G168S, G169D, and T302I) in the presence of both Ac-CoA (Figure 5A) and HMG-CoA (Figure 5B). Although no experimental measurement of enzymatic activity has been reported for the G168S and T302I variants (see Table 1 and Table 2), their behavior has been simulated as an exception due to their special position in the active site of the enzyme. Figure 5A shows that, for Y167C, G169D, and T302I (but not G168S), the position of the Ac-CoA substrate changes over time, moving away from the active center. This could explain the lack of enzymatic activity. The behavior of the four mutants in the presence of HMG-CoA resembles that of the wild-type enzyme, though it is somewhat less pronounced for Y167C. Changes in the active center can also directly affect activity, for example, by the addition of a second Cys in the area (Y167C) that could compromise the acylation reaction involving Cys166 or the substitution of small, hydrophobic amino acids such as Gly by larger and negatively charged amino acids such as Asp (G169D), which would change the geometry and electrostatic properties of the cavity, hindering or preventing the chemical reaction. The G168S variant behaves similarly to the wild-type protein in the presence of the Ac-CoA substrate, remaining in position after 500 ns of simulation (Figure 5C). Also, its behavior in the presence of the HMG-CoA product is similar to that of the wild-type protein, as shown in Figure 5B. Although no experimental enzymatic activity has been reported for this variant, the change in the geometric or charge conditions of the active center, caused by positioning a medium-sized, polar Ser instead of a small, hydrophobic Gly, could be the cause of the phenotype in patients due to incorrect enzyme function. In the case of the T302I variant, the replacement of a polar amino acid such as Thr by a hydrophobic one such as Ile causes a drastic change in the channel geometry and interaction with the Ac-CoA substrate, leading to a complete delocalization of the product (Figure 5D) after 500 ns of simulation. It cannot be ruled out that this change may also modify the position of the adjacent His301 of the catalytic triad, also causing an additional decrease in the catalytic efficiency of the active center.

To summarize the results for this group of residues, three of the four variants (Y167C, G169D, and T302I) show a delocalization of the Ac-CoA substrate throughout the simulation, offering an explanation for the lack of enzymatic activity and, perhaps, for the cause of the disease. In contrast, the G168S variant behaves in a manner that is not very different from the wild-type enzyme. No measurements of enzyme activity in vitro have been reported for this variant, so it is unclear if the similarity in behavior would correspond to measurable enzyme activity.

### 2.2. Residues Located near the Substrate Binding Site

**Figure 6:**
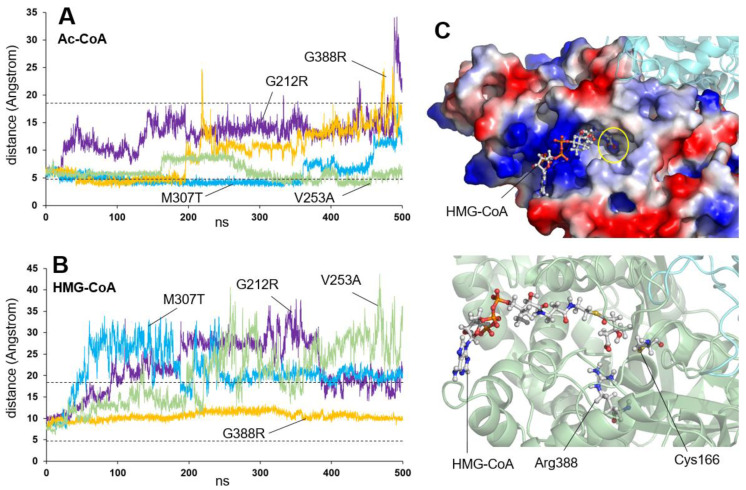
Stability of acetyl-CoA (Ac-CoA) or HMG-CoA molecules in the active center of mHS missense variants affecting residues located near the substrate binding site. (**A**,**B**) Plot showing the variation in the distance (in Angstrom) between the sulfur atom of the amino acid Cys166 and the sulfur atom of Ac-CoA (**A**) or HMG-CoA (**B**) molecules, measured over 500 ns of molecular dynamics simulation trajectory of the four variants G212R (violet), V253A (green), M307T (blue), and G388R (yellow). The dashed lines mark the average values measured for the wild-type protein (see Figure 1). (**C**) Representation of the HMG-CoA molecule after 500 ns of simulation for the G388R variant, stably located in the ligand site at the surface of the mHS protein. The yellow circle indicates the position of the blue-colored positive charge of Arg388 at the bottom of the tunnel (top). The negatively charged HMG group of the HMG-CoA molecule is positioned next to the positively charged Arg388 mutated residue and to the catalytic amino acid Cys166 (bottom).

The second group of residues is located close to the substrate channel (shown in blue in Figure 2). A simulation of their behavior in the presence of Ac-CoA (Figure 6A) and HMG-CoA (Figure 6B) was performed for the variants, for which an activity value had been obtained experimentally (Table 1 and Table 2): G212R, V253A, M307T, and G388R. The G212R variant’s behavior in the presence of Ac-CoA (Figure 6A) showed substrate delocalization in the first ns of simulation. This is probably due to the presence of a large, charged amino acid (such as Arg) at the Gly212 site, which explains its lack of activity in vitro. Later in the simulation, a similar behavior was observed with the G388R variant.

However, unlike the other three variants, the G388R variant exhibits behavior that is opposite to that of the wild type in the presence of HMG-CoA (see Figure 6B). Figure 6C (bottom) illustrates the position of HMG-CoA after 500 ns of simulation for the G388R variant, with the negatively charged HMG group positioned next to the positively charged Arg388 residue. Figure 6C (top) shows the HMG-CoA ligand firmly located on the surface of the protein within the substrate tunnel at the end of the molecular dynamics trajectory. The blue-colored positive charge due to the presence of Arg388 can be seen at the bottom of the tunnel. The lack of HMG-CoA product release after the enzymatic reaction could explain the G388R variant’s lack of activity. As the substrate tunnel is not free for a new Ac-CoA molecule after the previous reaction, the activity would be greatly diminished. This is the only case among the simulations performed in this study in which the HMG-CoA product behaves differently from the behavior observed in the wild-type protein.

The V253A variant exhibits a behavior similar to that of the wild-type protein. The Ac-CoA substrate oscillates in the vicinity of the active center without separating from it throughout the simulation (Figure 6A). Meanwhile, the HMG-CoA product rapidly delocalizes (Figure 6B), also similar to the wild-type protein. This behavior aligns with the experimentally determined enzyme activity value of 24% relative to the wild-type value. The difference between the two enzymes may be outside the prediction range of computational simulation techniques.

The M307T variant is also an intermediate case, as shown by the plots in Figure 6A,B. In the case of the amino acid Met307, this, in addition to forming part of the surface corresponding to the entrance mouth of the substrate channel, directly contacts Lys310, which intervenes in the stabilization of the phosphate group attached to the C3 carbon of the ribose of the substrates. It has been described that the amino acid Lys310 can be a substrate for an acetylation reaction [42], which would suggest a role in the regulation of the enzyme’s activity. The M307T variant would not only change the substrate channel geometry but could also laterally influence the regulation of the enzyme by acetylation, through distortion of the position of Lys310.

For the residues located near the substrate tunnel, a possible structural explanation for the lack of activity can also be inferred for three of the non-simulated variants: M381V, G89D, and E74K. The side chain of the amino acid Met381 is embedded in a narrow gap located between Gly212 and Gly388, both small, hydrophobic amino acids. In turn, Met381 directly contacts the amino acid Tyr282, which forms part of the substrate tunnel wall in close proximity to the active center. The exchange of either Gly for a large, positively charged residue such as Arg (G212R or G388R) or the exchange of Met381 for a shorter side chain amino acid such as Val (M381V) would distort the geometry of this interaction, causing substrate misplacement and thereby altering enzyme activity. The amino acid Gly89 localizes near the entrance of the substrate tunnel. Its change to Asp, a larger, negatively charged amino acid (G89D) is likely to affect the approach of substrates (with negative charges corresponding to acetyl, acetoacetyl, or phosphate groups present in their structures) to the cavity, hindering the initiation of the reaction. Finally, Glu74 contacts Lys83, involved in substrate localization. The E74K variant introduces a positive charge in place of the original negative charge, which will therefore distort the position of Lys83 and the localization of the substrate.

In summary, three types of behaviors can be observed in the analysis of this group of variants close to the substrate site. The first type, exhibited by the G212R and M307T variants, involves delocalization of the Ac-CoA substrate throughout the dynamics and explains the lack of enzymatic activity. The second type, represented by the V253A variant, exhibits behavior similar to the wild-type enzyme and corresponds well with 24.0% enzymatic activity (compared to the wild type). The third type, unique among all the variants studied, shows product retention in the G388R variant cavity due to the interaction between Arg388 and the HMG group of the HMG-CoA molecule.

### 2.3. Residues Located at the Homodimerization Surface

A third group of residues is formed by those in direct contact with residues of the opposite monomer or located in structures that stabilize the enzyme dimer’s geometry (residues in magenta in Figure 3). The crystallographic structure of the enzyme [7] shows that a correct dimer structure is critical for maintaining the geometry of the active center. In this structure, amino acid Tyr167 (see the Y167C variant above) is located next to the catalytic amino acid Cys166. It also contacts Gly159 of the opposite monomer through a water molecule [7]. Any variation in the position of these amino acids would be critical for the geometry of the active center, as they intervene in both the enzymatic activity and the correct dimerization of the enzyme.

**Figure 7:**
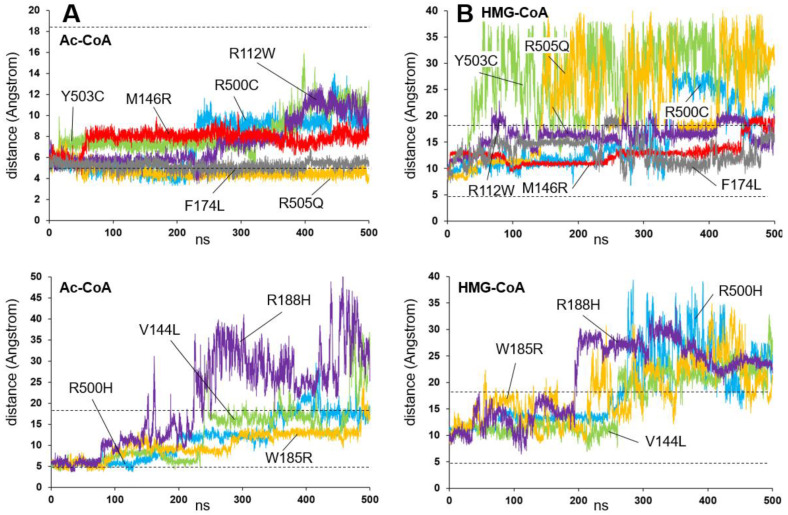
Location of acetyl-CoA (Ac-CoA) or HMG-CoA molecules in the active center of mHS missense variants affecting residues positioned at the homodimerization surface. (**A**,**B**) Plot showing the variation in the distance (in Angstrom) between the sulfur atoms of the amino acid Cys166 and the sulfur atoms of the Ac-CoA (**A**) or HMG-CoA (**B**) molecules, measured over 500 ns of the molecular dynamics simulation trajectory of the ten variants R112W, V144L, M146R, F174L, W185R, R188H, R500C, R500H, Y503C, and R505Q. For clarity, the plot has been divided into two, separating the variants with lower Ac-CoA substrate instability at the top and the variants with higher Ac-CoA instability at the bottom. Top: R112W (violet), M146R (red), F174L (gray), R500C (blue), Y503C (green), and R505Q (yellow); bottom: V144L (green), W185R (yellow), R188H (violet), and R500H (blue). The dashed lines mark the average values measured for wild-type protein (see Figure 1).

**Figure 8:**
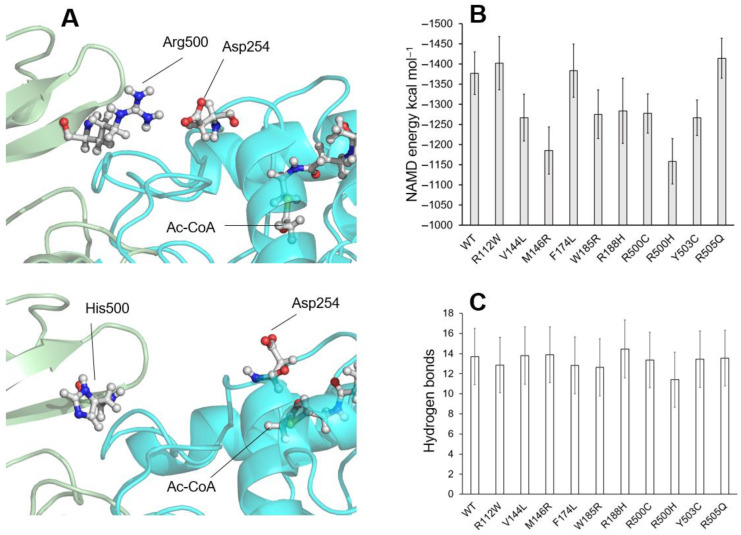
(**A**) Top: schematic showing the location of the wild-type Arg500 amino acid in one of the enzyme monomers bound to the Asp254 amino acid in the opposite monomer after 500 ns of molecular dynamics simulation. Bottom: the same scheme showing the position of the His500 amino acid (R500H variant), which has lost its interaction with the Asp254 amino acid in the opposite monomer after 500 ns of simulation. The position of a molecule of Ac-CoA is indicated. (**B**) Plot showing the mean +/− STD values of the interaction energy (in kcal mol^−1^) between the two enzyme monomers measured, using NAMD, over the last 200 ns of the molecular dynamics trajectories. (**C**) Plot showing the mean values +/− STD of the number of hydrogen bonds measured between the two monomers of the enzyme over the last 200 ns of the molecular dynamics trajectories.

To understand the influence of the variants located on the dimerization surface on the enzyme activity, the behavior of those whose activity has been determined experimentally (see Table 1 and Table 2): R112W, V144L, M146R, F174L, W185R, R188H, R500C, R500H, Y503C, and R505Q was simulated in the presence of the substrate Ac-CoA or HMG-CoA molecules (Figure 7). Interestingly, the accumulation of variants in the beta-sheet containing amino acids 500 to 505 brings together a remarkable cluster of variants (R500H, R500C, Y503C, and R505Q) to which we can add the variant R112W, located in an alpha helix running parallel to the beta-sheet. This structure rests, through the positively charged amino acid Arg500, on the surface of the opposite monomer, in contact with the negatively charged amino acid Asp254 (Figure 8A, top), thus stabilizing a dimerization that would be affected by the different variants in this local group of amino acids.

Figure 7A,B illustrates how the Ac-CoA substrate or the HMG-CoA positions, respectively, varies along the molecular dynamics trajectory for the ten variants. Additionally, the interaction energy between the two enzyme monomers was obtained using NAMD along the trajectories [43]. The energy values of each model during the first 300 ns of the simulation were discarded, assuming stabilization of the complexes during this time. The values from the remaining 200 ns were then averaged to obtain mean values +/− STD (Figure 8B).

Of the analyzed variants, F174L and R505Q stand out for their high substrate stability throughout the simulation (Figure 7A) and for their monomer–monomer interaction NAMD energy values, which are very similar to the energy values of the wild-type protein (Figure 8A). These results are consistent with the measured enzyme activity values for both variants (28.6% and 70.6%, respectively), which are significant fractions of the wild-type protein’s activity. The remaining variants exhibit a range of behaviors, from relatively stable, such as Y503 and R112W, to highly unstable, such as R188H and R500H. It is interesting to note that, in all cases, these behaviors correlate roughly with the NAMD energy values for the interactions between the monomers shown in Figure 8B.

To obtain a possible description of the reason for these different behaviors, the presence of hydrogen bonds between the two monomers of the enzyme was continuously measured during the last 200 ns of the simulation (Figure 8C). Although the values obtained do not show significant changes in the variants compared with the wild-type protein, there is a decrease of almost two points in the average value of the number of hydrogen bonds in the case of the R500H variant, which would correspond to the loss of interaction in the area surrounding this residue.

Figure 8A illustrates the location of the Arg500 amino acid in the wild-type protein after 500 ns of simulation. It forms a salt bridge with the Asp254 amino acid of the other monomer, thereby stabilizing dimerization. This position is compared with that of the His500 amino acid of the R500H variant after 500 ns of simulation (Figure 8A, bottom). In the R500H variant, the salt bridge is broken, which weakens the interaction between the monomers and causes the displacement of the adjacent structures.

In light of the simulation results, it does not appear that variants located in the area near the interface can cause the enzyme to lose its dimeric character. Rather, these mutations are more likely to cause changes in the interface structure that may be associated with changes in the active site geometry and, consequently, affect activity.

In the case of K243E, no experimental enzymatic activity has been reported for this variant [7], and so it has not been included in this molecular dynamics simulation study. Replacing a positively charged lysine residue with a negatively charged glutamic acid residue at the dimerization interface probably affects the protein’s enzymatic activity. Additionally, Lys243 belongs to a motif surrounding Ser248 and Tyr250, which have been described as substrates for phosphorylation [42]. Therefore, replacing Lys243 with Glu is also likely to affect enzyme activity regulation by phosphorylation.

As a summary of the behavior exhibited by the amino acids located in areas close to the interface between monomers, it is interesting to note that all of them, except for two (see below), cause the delocalization of the Ac-CoA substrate from the active center throughout the simulation, thus providing an explanation for the lack of activity. Further studies analyzing in detail the propagation of instability caused by residues at the interface towards the substrate cavity and active site would be interesting.

The F174L and R505Q variants exhibit different behaviors. These variants have a relationship with the substrate and product that is very similar to that of the wild-type enzyme. This is consistent with the measured in vitro enzymatic activities of 28.6% and 70.6% for the two variants, respectively.

### 2.4. Residues Located at Other Positions

This group consists of variants involving amino acids distant from the active center or dimerization surface. These variants may be more related to structural perturbations in the mature protein or during folding, which could lead to decreased or lost activity.

**Figure 9:**
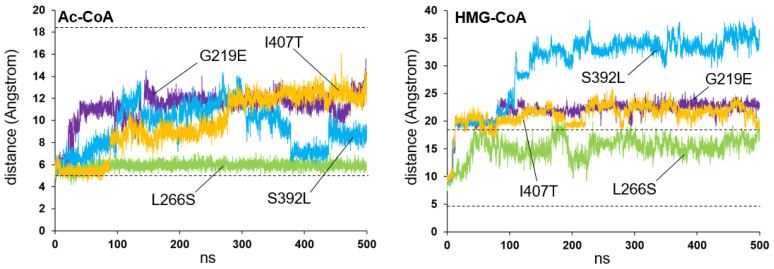
Analysis of the stability of acetyl-CoA (Ac-CoA, left) and HMG-CoA (right) molecules in the active center of mHS missense variants, affecting residues positioned distant from the active center or dimerization surface. Plots showing the variation in the distance (in Angstrom) between the sulfur atom of the amino acid Cys166 and the sulfur atom of the Ac-CoA or HMG-CoA molecules, measured over 500 ns of molecular dynamics simulation trajectory of the four variants: G219E (violet), L266S (green), S392L (blue), and I407T (yellow). The dashed lines mark the average values measured for wild-type protein (see Figure 1).

Figure 9 shows the stability of the Ac-CoA or HMG-CoA positions along the molecular dynamics trajectory in the four analyzed structures: G219E, L266S, S392L, and I407T. All of the structures exhibit some Ac-CoA substrate instability, except for the L266S variant, which behaves similarly to the wild-type protein. In the case of the HMG-CoA product, the four variants exhibit a similar behavior to the wild-type protein.

While establishing a cause-and-effect relationship with this group of variants is difficult, it is reasonable to assume that amino acid changes introduce distortions in beta sheets or alpha helices that would most likely alter their geometry. This alteration would then propagate to the rest of the protein, affecting its enzymatic activity.

**Figure 10:**
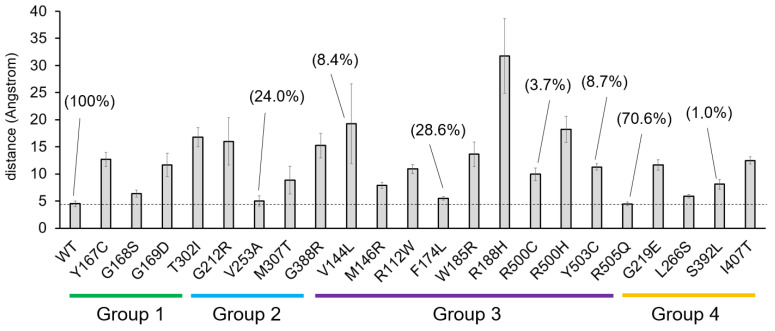
Plot summarizing the results obtained for the behavior of the acetyl-CoA (Ac-CoA) substrate in all the variants analyzed throughout this study. The average distance (in Angstroms) +/− STD between the sulfur atom of the amino acid Cys166 and the sulfur atom of the Ac-CoA molecule is shown, measured during the last 200 ns of the molecular dynamics trajectories. Group 1: residues located close to the active center; Group 2: residues located near the substrate binding site; Group 3: residues located at the homodimerization surface; Group 4: residues located at other positions. The enzymatic activity of variants for which it is detectable and has been experimentally measured is indicated as a percentage of the activity of the wild type (WT, 100%).

Figure 10 summarizes the average distance measurement between the sulfur atom of amino acid Cys166 and the sulfur atom of the Ac-CoA molecule during the last 200 ns of the molecular dynamics trajectories. This measurement illustrates the “final state” after the simulation. Interestingly, many variants for which a specific enzymatic activity value has been determined as a measurable fraction of wild-type activity also show distance values that are similar to the wild-type enzyme. Thus, the Ac-CoA substrate would be in a favorable position for enzyme action with the V253A, F174L, and R505Q variants, which have experimentally measured activities of 24.0%, 28.6%, and 70.6%, respectively, compared with the wild type. However, this is not always the case, as seen with the V144L and Y503C variants.

Molecular dynamics computational simulations of macromolecular structure movements are tools that rationalize the effects of certain protein sequence variants on function and relationships with other molecules at the atomic level. To our knowledge, no previous work has studied the effect of different mHS variants on the reaction they catalyze at the atomic or biophysical levels. For this reason, we believe that molecular dynamics simulation techniques are one of the few available avenues for approaching an atomic or molecular rationalization of the phenotypic effects of each variant behavior.

As shown in the present work, molecular dynamics simulations reasonably align with experimental results from enzyme activity measurements in most cases. This reaffirms the possibility of applying them to new mHS mutations that may be discovered in the future, as well as for the future analysis of correlation between the movements of atoms surrounding the different amino acid variants and how this variation is transmitted at a distance to the active center.

However, many questions remain about the effects of variants for which no variation in their interaction with substrates has been found or even questions about the nature of their interaction with intermediate substrates. For example, the binding efficiency of substrates and/or products could be evaluated by measuring dissociation constants through isothermal titration calorimetry. Future work using biophysical techniques to analyze enzyme–substrate interactions may provide answers to these questions.

## 3. Materials and Methods

The 3D structure of the human mitochondrial HMG-CoA synthase enzyme (Uniprot ID: P54868) in its binary product complex with HMG-CoA, was obtained from the Protein Data Bank (2WYA; [7]). The initial model of the enzyme interacting with its substrate, acetyl-CoA, was generated by replacing the relevant atoms in the co-crystallized HMG-CoA molecule. The topology parameters of both acetyl-CoA and HMG-CoA in their protonated state at a pH of 7 were generated using ACPYPE (AnteChamber PYthon Parser interfacE; [44,45]).

The Swiss-Model server (http://swissmodel.expasy.org, accessed on January 2025) was used to build initial models of homozygous enzyme variants with structural qualities within the range of those accepted for homology-based structures. The different variants are simulated in homozygosity to align the results to laboratory enzyme measurements, which are performed using enzymes synthesized in vitro and purified, and are therefore homozygous. This also corresponds to the presumed state of the enzymes in patients, since the disease is autosomal recessive.

Once modeled, the interaction of the wild-type or variant enzymes with the substrate, acetyl-CoA, or the final product, HMG-CoA, was simulated over a period of 500 ns of unrestrained molecular dynamics (MD) simulation using the Amber18 package (University of California-San Francisco, CA, USA), essentially as previously described [46,47]. In brief, 3D models were first solvated with a periodic octahedral pre-equilibrated solvent box using the LEaP module of Amber18, with 12 Å as the shortest distance between any atom in the protein subdomain and the periodic box boundaries. Molecular dynamics simulations were conducted employing the Particle Mesh Ewald (PME) method for non-bonded interactions with a cutoff distance of 8 Å. The temperature was regulated through the Langevin thermostat, maintaining a fixed temperature of 297 K with a collision frequency of 1 ps. Hydrogen bond constraints were implemented using the SHAKE algorithm, enabling a simulation timestep of 2 fs. To uphold NPT conditions (constant number of particles, pressure, and temperature), the pressure coupling was managed by a Monte Carlo Barostat set to 297 K and 1 bar. The initial model structures underwent 10,000 cycles of energy minimization, followed by a 1 ns restrained equilibration phase, smoothly raising the temperature to 297 K, after which restraints were gradually removed over 10 ns. Subsequently, each system (32 in total) was subjected to a 500 ns long free-MD-production phase. Trajectories, including distance measurements between the sulfur atom of the catalytic amino acid Cys166 and the sulfur atom of the Ac-CoA or HMG-CoA molecules, as well as number of hydrogen bonds between enzyme monomers, were analyzed using cpptraj [48] and VMD [49].

To assess whether the simulated systems had reached sufficient stability during molecular dynamics trajectories, RMSD values were calculated using VMD (Supplementary Figure S1). In all cases, the variation in RMSD values from nanosecond 100 to nanosecond 500 of the trajectories was less than 1.5 Angstrom, indicative of structural stability.

The evaluation of nonbonding energy contributions of the surface interaction between the two enzyme monomers along the simulations was performed using the NAMD Energy Plugin of VMD (NAMD v.2.14) [43]. Figures were generated with Pymol (https://pymol.org).

## Figures and Tables

**Table 1:** mHS cDNA variants.

cDNA	Protein	* Enzyme Activity	Reference (s)
chr1 del. 120, 310, 549-120, 312, 114, GRCh37/hg19	deletion of exon 1 and part of intron 1	-	[15]
c.1-?_104+?del	deletion of exon 1	-	[11,16]
c.34dupA	p.L14Tfs59 *	-	[17]
c.100C>T	p.Q34 *	-	[18]
c.104G>T	p.R35M	-	[19]
c.130_131insC	p.L44Pfs * 29	-	[20]
c.160G>A	p.V54M	-	[17,21,22]
c.167T>A	p.I56N	-	[7]
c.181_182ins22	p.V61Afs * 19	-	[21]
c.220G>A	p.E74K	-	[17]
c.252T>G	p.Y84 *	-	[7]
c.266G>A	p.G89D	-	[23]
c.334C>T	p.R112W	Inactive	[13,15]
c.407A>G	p.D136G	-	[15,17]
c.409A>T	p.K137 *	-	[24]
c.422T>A	P.V141D	-	[17]
c.430G>T	p.V144L	8.4%	[13]
c.431_432del	p.V144Sfs * 11	-	[11]
c.437T>C	p.M146R	Inactive	[13]
c.476G>T	p.G159V	-	[25]
c.500A>G	p.Y167C	Inactive	[9]
c.502G>A	p.G168S	-	[11]
c.506G>A	p.G169D	Inactive	[11,16]
c.512C>T	p.A171V	-	[26]
c.520T>C	p.F174L	28.6%	[11,27,28,29]
c.520T>G	p.F174V	-	[30]
c.553T>C	p.W185R	Inactive	[11]
c.559+1G>T	splicing variant	-	[15,17,21,31]
c.563G>A	p.R188H	Inactive	[31,32]
c.616C>T	p.R206C	-	[31]
c.634G>A	p.G212R	Inactive	[9,11,33,34]
c.648G>T	p.M216I	-	[35]
c.656G>A	p.G219E	Inactive	[14]
c.695G>T	p.G232V	-	[11]
c.682C>T	p.R228 *	-	[15]
c.697A>G	p.T233A	-	[7]
c.704T>C	p.M235T	-	[14]
c.717T>G	p.Y239 *	-	[17]
c.719A>C	p.D240A	-	[17]
c.727A>G	p.K243E	-	[7]
c.758T>C	p.V253A	24.0%	[14,36]
c.788delT	p.L263Cfs36 *	-	[17]
c.797T>C	p.L266S	Inactive	[11,16]
c.821G>A	p.R274H	-	[17]
c.830T>A	p.I277D	-	[21]
c.847C>T	p.Q283 *	-	[11]
c.850+1G>A	splicing variant	-	[15]
c.851-2A>C	splicing variant	-	[37]
c.862C>T	p.R288 *	-	[37]
c.905C>T	p.T302I	-	[26]
c.920T>C	p.M307T	Inactive	[32]
c.965C>G	p.S322 *	-	[30]
c.1016+1G>A	splicing variant	-	[15,33,34]
c.1017-2A>G	splicing variant	-	[17]
c.1078T>C	p.S360P	-	[11]
c.1090T>A	P.F364I	-	[15]
c.1118A>T	p.Y373F	-	[38]
c.1141A>G	p.M381V	-	[24]
c.1156_1157insC	p.L386Pfs * 73	-	[20]
c.1162G>A	p.G388R	Inactive	[11,12,16]
c.1175C>T	p.S392L	1.0%	[14,36]
c.1187+1G>C	splicing variant	-	[17,25,35]
c.1201G>T	p.E401 *	-	[17,31]
c.1220T>C	p.I407T	Inactive	[11,12,16]
c.1259T>C	p.F420S	-	[31]
c.1270C>T	p.R424 *	-	[28,39]
c.1279C>T	p.Q427 *	-	[31]
c.1347_1351delAGCCT	p.A450Pfs * 7	-	[31]
c.1349delA	p.N465Tfs1 *	-	[17,40]
c.1465delA	p.T489Lfs * 55	-	[18]
c.1480C>T	p.R494 *	-	[27]
c.1498C>T	p.R500C	3.7%	[14]
c.1499G>A	p.R500H	Inactive	[9,14]
c.1502G>C	p.R501P	-	[21,41]
c.1502G>A	p.R501Q	-	[15,17,27,40]
c.1508A>G	p.Y503C	8.7%	[11]
c.1514G>A	p.R505Q	70.6%	[11,13,16]

Variants described in the cDNA of the mHS protein, which are considered responsible for mHS deficiency. (*) Reported enzyme activity referred to wild type (100%); (-): not assayed; (Inactive): activity below the assay detection limits and 100% loss of function assumed.

**Table 2:** mHS variants simulated using molecular dynamics.

Group	Variant	* Enzyme Activity
1. Residues located close to the active center	p.Y167C	Inactive
	p.G168S	-
	p.G169D	Inactive
	p.T302I	-
2. Residues located near the substrate binding site	p.G212R	Inactive
	p.V253A	24.0%
	p.M307T	Inactive
	p.G388R	Inactive
3. Residues located at the homodimerization surface	p.V144L	8.4%
	p.M146R	Inactive
	p.R112W	Inactive
	p.F174L	28.6%
	p.W185R	Inactive
	p.R188H	Inactive
	p.R500C	3.7%
	p.R500H	Inactive
	p.Y503C	8.7%
	p.R505Q	70.6%
4. Residues located at other positions	p.G219E	Inactive
	p.L266S	Inactive
	p.S392L	1.0%
	p.I407T	Inactive

mHS variants simulated using molecular dynamics, grouped according to their location in the protein structure. (*) Reported enzyme activity referred to wild type (100%); (-): not assayed; (Inactive): activity below the assay detection limits and 100% loss of function assumed.

## Data Availability

The data analyzed during this study are included in this published article. Additional supporting data are available from the corresponding authors upon reasonable request.

## Supplementary Materials

The following supporting information can be downloaded at https://www.mdpi.com/article/10.3390/ijms26178266/s1.
