# Supplementary material for: Ligand–Enzyme Interaction Modeling of Missense Variants Implicated in Mitochondrial HMG-CoA Synthase Deficiency
Source: Int J Mol Sci. 2025 Aug 26;26(17):8266. doi: 10.3390/ijms26178266 (PMC12428298; doi:10.3390/ijms26178266)
Supplement: Supplementary file 1 [file ijms-26-08266-s001.zip › ijms-3788880-supplementary.pdf]

Ligand-enzyme interaction modeling of missense variants im-plicated in mitochondrial HMG-CoA synthase deficiency.

María Arnedo, David Ros-Pardo, Beatriz Puisac, Cristina Lucia-Campos, Marta Gil-Salvador, Ana Latorre-Pellicer, Íñigo Marcos-Alcalde, Juan Pié & Paulino Gómez-Puertas.

Supplementary Figure S1. Root mean square deviation (RMSD) values, in Angstrom, were measured along molecular dynamics trajectories (500 ns). The different variants analyzed have been grouped according to the classification in Table 2. In all cases, the variation in RMSD values from nanosecond 100 to nanosecond 500 of the trajectories was less than 1.5 Angstrom, indicative of structural stability.

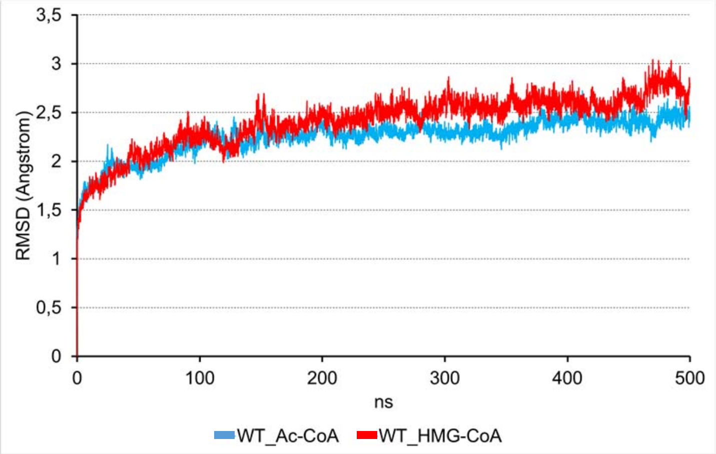

mHS (wild-type). RMSD values. Ac-CoA and HMG-CoA

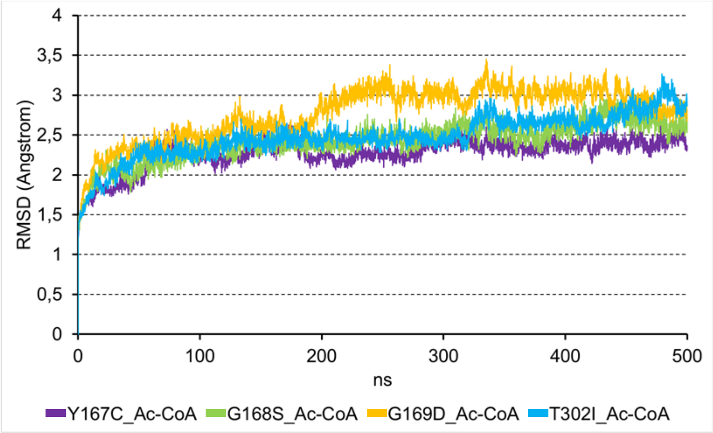

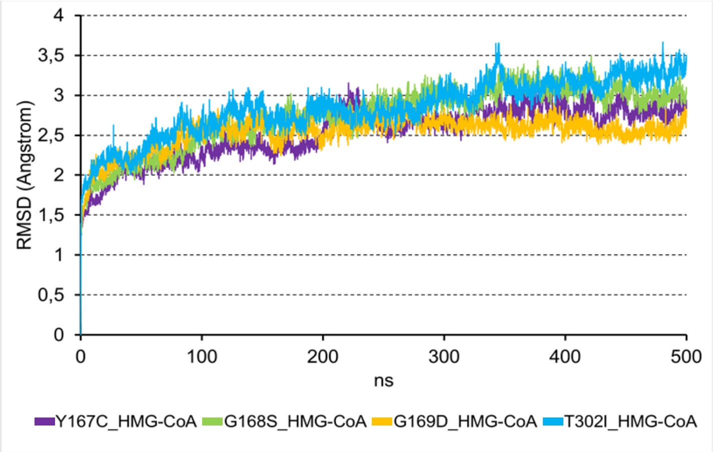

Group 1. Residues located close to the active center.
RMSD values. Ac-CoA (left) and HMG-CoA (right)

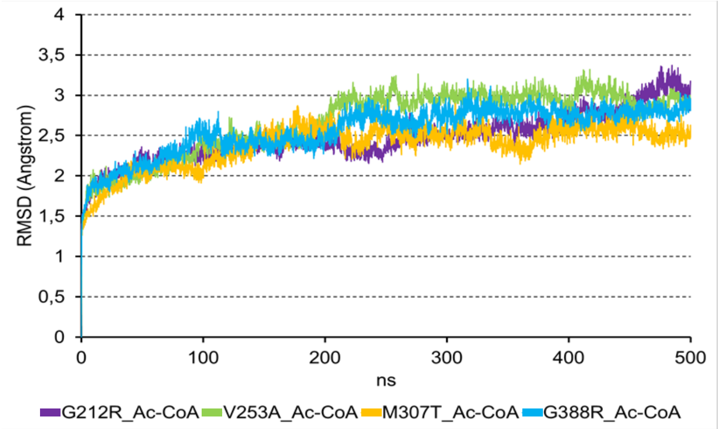

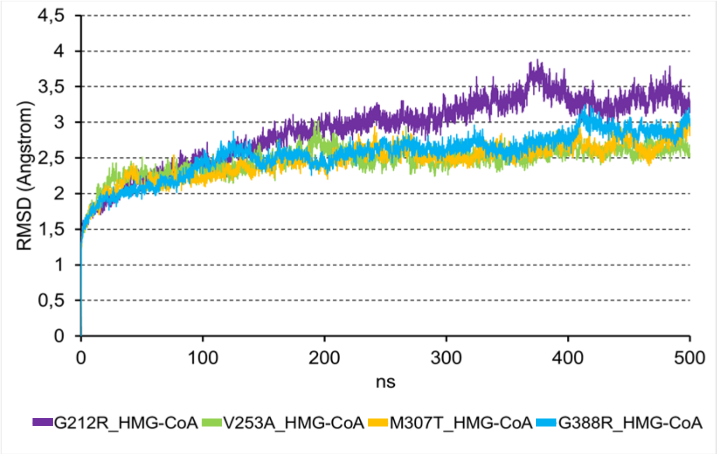

Group 2. Residues located near the substrate binding site.
RMSD values. Ac-CoA (left) and HMG-CoA (right)

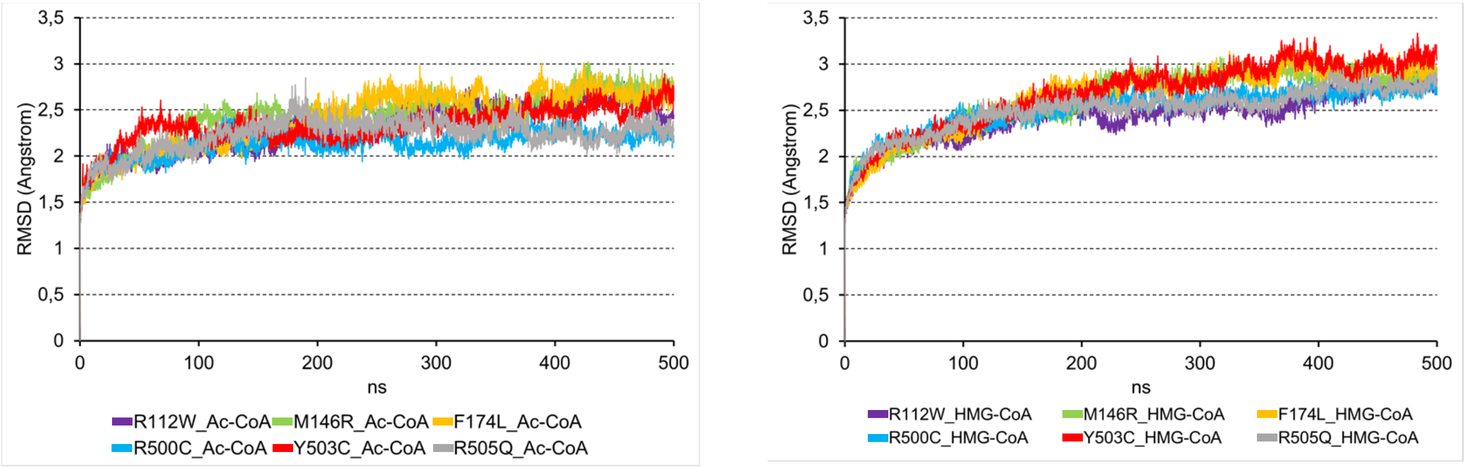

Group 3. Residues located at the homodimerization surface.
RMSD values. Ac-CoA (left) and HMG-CoA (right)

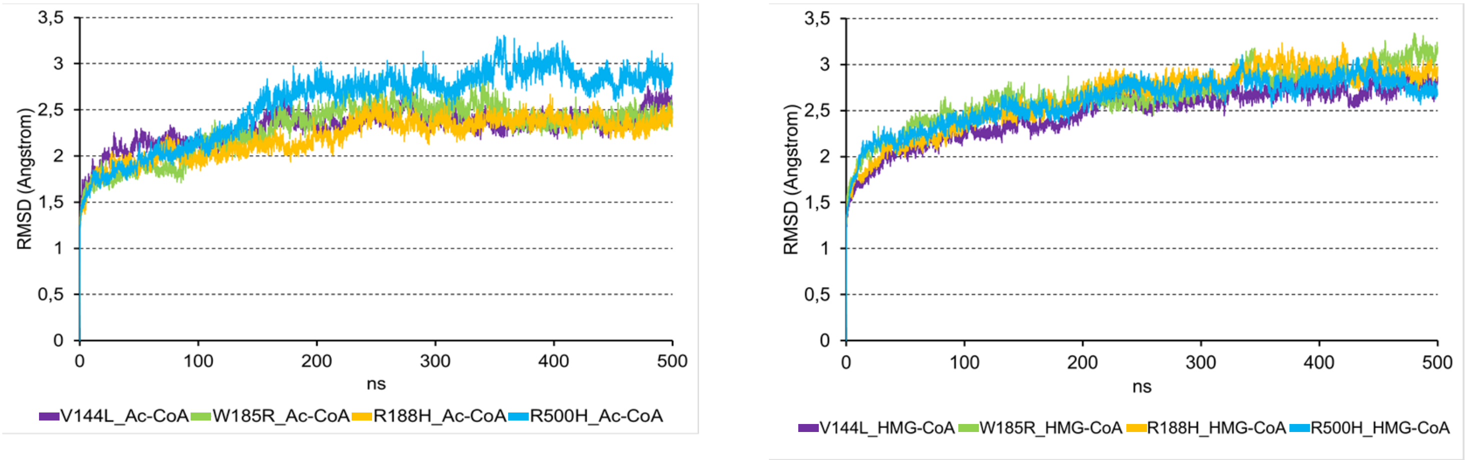

Group 3. Residues located at the homodimerization surface.
RMSD values. Ac-CoA (left) and HMG-CoA (right)

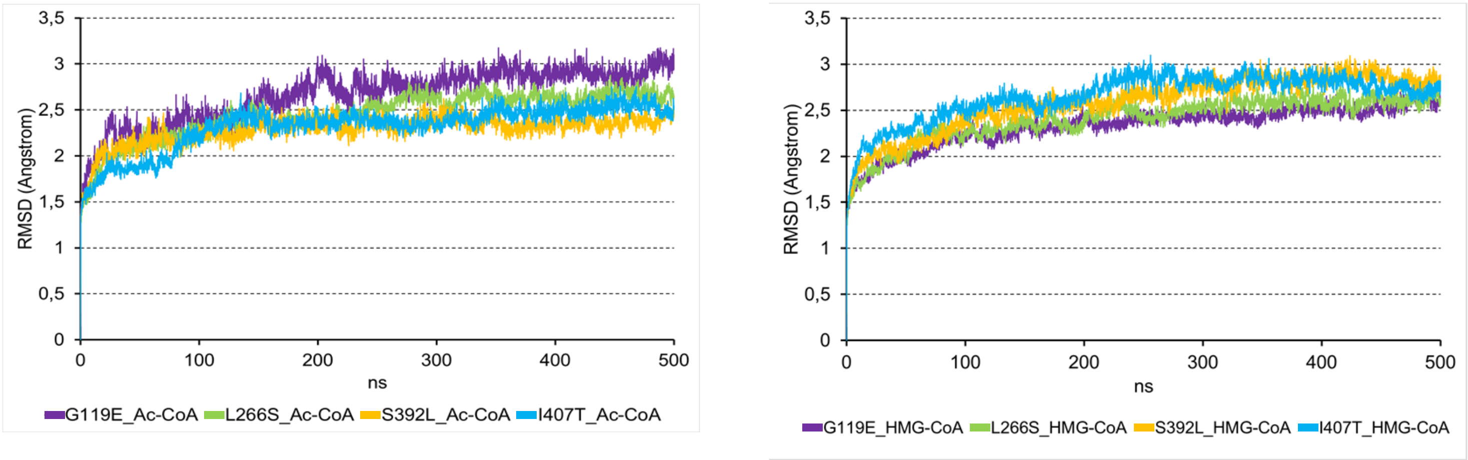

Group 4. Residues located at other positions.
RMSD values. Ac-CoA (left) and HMG-CoA (right)
